# Exploring the Psychological Processes That Underlie Interpersonal Forgiveness: Replication and Extension of the Model of Motivated Interpersonal Forgiveness

**DOI:** 10.3389/fpsyg.2020.02107

**Published:** 2020-10-08

**Authors:** Leigh Anne N. Donovan, Joseph R. Priester

**Affiliations:** ^1^Marketing Department, Lubin School of Business, Pace University, New York, NY, United States; ^2^Department of Marketing, Marshall Business School, University of Southern California, Los Angeles, CA, United States; ^3^Department of Psychology, Dornsife College of Letters, Arts, and Sciences, University of Southern California, Los Angeles, CA, United States

**Keywords:** forgiveness, motivated reasoning, empathy, relationship closeness, Model of Motivated Interpersonal Forgiveness

## Abstract

When, why, and how does interpersonal forgiveness occur? These questions guided recent research that compared the relative abilities of empathy versus motivated reasoning models to account for the influence of relationship closeness on interpersonal forgiveness. Consistent support was provided for the Model of Motivated Interpersonal Forgiveness. This model hypothesizes that, following relationship transgressions, relationship closeness leads to a desire to maintain a relationship. Desire to maintain a relationship leads to motivated reasoning. And motivated reasoning fosters interpersonal forgiveness. The goal of the present research was to examine two concerns that emerged from the initial support for the Model of Motivated Interpersonal Forgiveness. First, were the measures of motivated reasoning and interpersonal forgiveness conflated, thus reducing the potential for empathy to account for interpersonal forgiveness? Second, did the analytic estimation used reduce the power to detect the mediational role of empathy? The present research examined these questions. When motivated reasoning was measured by thought listings (in addition to the original questionnaire items) and when the analytic estimation provided greater power, the Model of Motivated Interpersonal Forgiveness was replicated.

## Introduction

We are a social species, surrounded by and connected to others: relationships give our lives meaning and sustenance (Aristotle. [350 BCE], 2009; Clark and Grote, 2013; Aronson and Aronson, 2018; Murray and Holmes, 2011). As humans, we are bound at some point to slight, disappoint, hurt, and even betray the people in our lives; be they family, close friends, or acquaintances (e.g., Rusbult et al., 1991; Fincham et al., 2004; Keiningham et al., 2010). And yet, these relationships typically endure, continuing past such transgressions. One important way they do so is through the power of forgiveness.

But what leads to forgiveness? Is it the result of one’s ability to understand and experience the feelings of others? Or does it emerge instead from the story that we construct by which to understand the offense? More specifically, what are the psychological processes that underlie, and give rise to, interpersonal forgiveness? The goal of this article is to deepen our understanding of these processes by more thoroughly testing the recently proposed (and empirically supported) Model of Motivated Interpersonal Forgiveness (Donovan and Priester, 2017) in comparison to the empathy model of interpersonal forgiveness (McCullough et al., 1997, 1998).

The Model of Motivated Interpersonal Forgiveness was advanced to understand when, why, and how interpersonal forgiveness unfolds. In brief, the Model of Motivated Interpersonal Forgiveness hypothesizes a sequential mediation model. Interpersonal forgiveness occurs when one feels close to a transgressor because such closeness leads to a desire to maintain the relationship, which leads to motivated reasoning. And it is motivated reasoning that fosters forgiveness. In this article, we provide an explanation and review evidence in support of the Model of Motivated Interpersonal Forgiveness, examine two concerns stemming from the Donovan and Priester (2017) studies, and report the results of an empirical study that explores these questions.

## The Scientific Study of Interpersonal Forgiveness

Prior to the 1990s, the majority of published work on forgiveness was within the domains of religion, philosophy, and psychiatry. Thanks to the seminal work of the pioneering researchers Michael McCullough, Carol Rusbult, and Everett Worthington (among others), interpersonal forgiveness came into prominence as a topic of scientific study and has grown since (e.g., Rusbult et al., 1991; McCullough et al., 1997, 1998, 2013). For example, a search on the Web of Science reveals that prior to 1990 there were fewer than 125 articles published that touched upon forgiveness. Since 1991, more than 5,000 such articles have been published^1^.

The psychological research on interpersonal forgiveness has generally fallen within one of two theoretical perspectives. While both perspectives posit the critical importance of relationship closeness in forgiveness, they differ as to the hypothesized process that underlies the influence of relationship closeness on forgiveness. The more dominant perspective conceptualizes interpersonal forgiveness as the result of an individual’s empathy for the person who transgressed (McCullough et al., 1997, 1998). The other perspective conceptualizes forgiveness to be the result of the story that one constructs to make sense of a transgression, a process referred to as motivated reasoning (Donovan and Priester, 2017).

## Antecedents of Interpersonal Forgiveness

### Relationship Closeness

What is known from the literature on forgiveness? Relationship closeness matters! Relationship closeness, in its various conceptualizations and operationalizations, is the most robust and frequently explored antecedent of interpersonal forgiveness (Fehr et al., 2010). The more committed (e.g., Finkel et al., 2002), satisfied (e.g., Allemand et al., 2007), trusting (e.g., Rempel et al., 2001), and connected (e.g., McCullough et al., 1998) a relationship, the more likely that one is to forgive a transgression by that partner. But what underlies relationship closeness’s influence on forgiveness? This is the question about which the two theoretical perspectives differ.

### Empathy

Beginning in the 1990s, empathy came to be perceived as a critical psychological component in interpersonal relationships. Empathy was implicated in a variety of prosocial behaviors (e.g., Eisenberg and Miller, 1987; Batson, 1990, 1991; Eisenberg and Fabes, 1990), as well as relationship well-being (e.g., Davis and Oathout, 1987; Rusbult et al., 1991). Empathy has been defined in a number of ways (Kunyk and Olson, 2001; Cuff et al., 2016), but all rely upon the notion that empathy is an emotion toward another, typically associated with such feelings as sympathy, compassion, and tenderness (McCullough et al., 1997).

**FIGURE 1:**
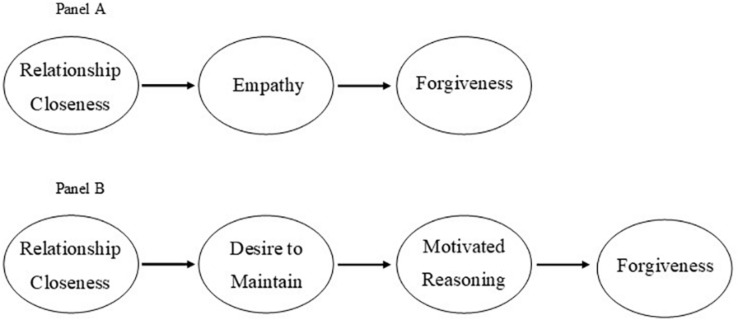
The Empathy Model pf Int erpersonal Forgiveness **Panel A** and the Model of Motivated Interpersonal Forgiveness **Panel B**.

One of the first and arguably most influential programs of research to explore interpersonal forgiveness was developed by McCullough and Worthington (1994) and McCullough et al. (1997, 1998). This model posits that interpersonal forgiveness comes about because of empathy for the transgressor: The more one feels empathy for another, the more one is likely to forgive. Indeed, empathy is inextricably linked to forgiveness in this model, in which interpersonal forgiveness is defined as an empathy-facilitated set of motivational changes (p. 321, McCullough et al., 1997). Indeed, empathy is hypothesized to be the most powerful antecedent of interpersonal forgiveness. It is hypothesized that although other variables (such as relationship closeness and motivated reasoning) may be associated with interpersonal forgiveness, “the associations of such variables with forgiving tend to be relatively small after controlling the indirect effects that they have on forgiving by means of their effects on empathy” (p. 1588, McCullough et al., 1998). In other words, empathy should mediate the influence of other constructs on interpersonal forgiveness. As such, empathy is conceptualized to be the most proximal mediator of interpersonal forgiveness (p. 1587, McCullough et al., 1998). Support for this model has been provided across many studies and articles, conducted both by McCullough et al. (1997, 1998) and others (Zechmeister and Romero, 2002; Paleari et al., 2005). This model is presented in Figure 1A.

### Motivated Reasoning

At the most basic, Kunda (1990) argued that one’s wish, desire, or preference can bias cognitive processes such that one’s understanding of a person, event, or object are consistent with one’s desire. The more one desires, the more one is likely to retrieve memories and/or construct beliefs that align with one’s desired outcome. Thus, desire may lead to a distorted understanding of the nature, causes, and likelihood of various events. That is, one constructs a story that allows oneself to arrive at the desired outcome.

At the same time as the emergence of the empathy model of interpersonal forgiveness, several different research programs began to provide evidence for the importance of motivated reasoning in interpersonal relationships. One such program, spearheaded by Carol Rusbult and her students, examined the influence of relationship commitment on accommodation (how an individual responds to a partner’s “breaches of good behavior,” p. 53; Rusbult et al., 1991). This research found, in part, that relationship commitment influenced accommodation because of a person’s explanation for a partner’s behavior. For example, Finkel et al. (2002) found that one’s attributions (i.e., motivated reasoning) following a transgression mediate the influence of relationship commitment on forgiveness (see also, Fehr et al., 2010). Independent of Rusbult et al. (1991) found robust evidence that one’s interpretation of a partner’s behavior is critical in relationship maintenance. For example, they found that individuals are able to cognitively transform a partner’s negative actions into positive narratives. Murray and Holmes referred to this process as “positive illusions.” Positive illusions lead to greater relationship resilience, which in turn leads to stronger positive illusions, thus creating a virtuous cycle^2^.

### Forgiveness

What is forgiveness? Although seemingly a basic question, there is not a single agreed upon definition of forgiveness (Worthington, 1998). The closest to such a definition would be that forgiveness occurs within the context of a relationship following a transgression (Fincham, 2000; Kearns and Fincham, 2004; Hannon et al., 2010) and is a process that takes place over time from which a “suite of prosocial changes” toward the transgressor emerges (491; McCullough et al., 2007; Fehr et al., 2010).

These changes are often defined in terms of revenge and avoidance (McCullough et al., 2007)^3^. That is, forgiveness is evidenced by reduced feelings of revenge and/or avoidance. Of note is that researchers typically include a measure of an individual’s own understanding of forgiveness by including a question as to whether that person has forgiven the transgressor (e.g., Girard and Mullet, 1997; Berry et al., 2001; Fincham and Beach, 2002; Karremans et al., 2003, 2005; Exline et al., 2004; Zechmeister et al., 2004; Kearns and Fincham, 2005; Finkel et al., 2007; Green et al., 2008; Hannon et al., 2010).

## The Model of Motivated Interpersonal Forgiveness

Given the independence of these two research streams, it is not surprising that few studies compared the two explanations for interpersonal forgiveness. And yet the question remained, did empathy and/or motivated reasoning underlie interpersonal forgiveness? To directly test this question, Donovan and Priester (2017) integrated an additional antecedent with motivated reasoning in order to derive the Model of Motivated Interpersonal Forgiveness. This additional antecedent is the desire to maintain the relationship.

### Desire to Maintain the Relationship

In much of their research, Rusbult and colleagues used interpersonal commitment as their focal construct. In one study, Finkel et al. (2002) explored the bases of such commitment and their relative influence on forgiveness. They found that both psychological attachment, which represents the extent to which one feels connected to another (and to which we refer as relationship closeness), and intent to persist, which represents the extent to which one desires and intends to maintain the relationship (and to which we refer as desire to maintain the relationship), both significantly predicted forgiveness individually. However, simultaneous analyses provided evidence that the influence of relationship closeness on forgiveness was mediated by the desire to maintain the relationship.

Desire to maintain the relationship provides a potentially critical step in the interpersonal forgiveness process in that it may help elucidate *why* relationship closeness fosters interpersonal forgiveness. Relationship closeness may foster forgiveness precisely because of one’s desire to maintain the relationship. If so, then desire to maintain the relationship may provide the underlying power of relationship closeness. However, although one may forgive because of one’s desire to maintain the relationship, such forgiveness requires justification. Lack of such justification would lead to a threat to the self and feelings of discomfort (viz., cognitive dissonance; see, for example, Aronson, 1969). Fortunately, motivated reasoning can provide such justification. One can continue a transgressed relationship one desires to maintain without threat to the self because of the story that one constructs to understand the transgression. That is, motivated reasoning provides the *how* (or process) by which one can justify continuing a relationship with the person who has harmed us yet with whom we desire to maintain the relationship.

### Desire to Maintain the Relationship and Motivated Reasoning as a Process Underlying Interpersonal Forgiveness

Donovan and Priester (2017) integrated the desire to maintain the relationship and motivated reasoning to arrive at the Model of Motivated Interpersonal Forgiveness. This model hypothesizes that (a) relationship closeness leads to a desire to maintain the relationship, (b) desire to maintain the relationship leads to motivated reasoning, and (c) motivated reasoning leads to interpersonal forgiveness. Such a model addresses *when* (close interpersonal relationships), *why* (desire to maintain the relationship), and *how* (motivated reasoning) interpersonal forgiveness may emerge. This model is depicted in Figure 1B.

### Empirical Support

Donovan and Priester (2017) examined the relative efficacy of the empathy model and the Model of Motivated Interpersonal Forgiveness across three studies. Two of the studies relied upon the individual’s recollection of a specific transgression, and the third used a hypothetical scenario in which that person is let down by another. Studies 2 and 3 measured relationship closeness, empathy, desire to maintain the relationship, motivated reasoning, and forgiveness.

**FIGURE 2:**
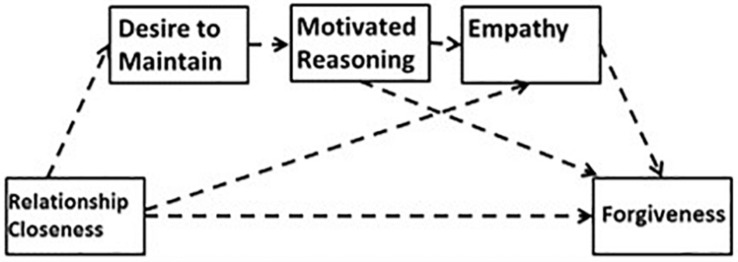
Analytic estimation used in Donovan and Priester (2017).

In order to test between the two perspectives, Donovan and Priester (2017) simultaneously estimated a combination of possible mediational paths by bootstrap OLS regression analyses (Hayes, 2013). The estimation allowed for the possibility that the influence of relationship closeness on forgiveness was mediated by (a) desire to maintain the relationship through motivated reasoning (representing the Model of Motivated Interpersonal Forgiveness), and/or (b) empathy (representing the empathy model of forgiveness). The specific ordering of the mediators allowed for empathy to serve as the most proximal mediator of forgiveness, as suggested by McCullough et al. (1998). This estimation allowed for one, both, or neither of the paths to emerge as significant. The estimation is presented in Figure 2. The paths relevant to the two models are depicted by the arrows among the key variables. Of note, however, is that all possible paths (e.g., relationship closeness to desire to maintain the relationship to forgiveness) were simultaneously tested in this order.

Across all three studies, the results revealed that the Model of Motivated Interpersonal Forgiveness was able to significantly predict interpersonal forgiveness, whereas the empathy model of forgiveness was not. Specifically, the analyses revealed that the mediational path of relationship closeness → desire to maintain the relationship → motivated reasoning → forgiveness emerged as significant, whereas the other possible paths did not. None of the paths that included empathy emerged as significant when simultaneously estimated with the Model of Motivated Interpersonal Forgiveness^4^. These results provide support for the notion that the psychological processes underlying interpersonal forgiveness are better explained by the Model of Motivated Interpersonal Forgiveness than by an empathy model.

This investigation also shed light on the nature of motivated reasoning. In the third study, a wide array of questions was used in order to capture motivated reasoning. When all of the questions were combined to create one measure, that measure emerged as the most proximal antecedent to forgiveness. Additional analyses revealed that the influence of this measure of motivated reasoning was driven by one’s perception of the transgressor and one’s expectation of future behavior.

## Remaining Questions

### Motivated Reasoning and Forgiveness

Recall that Donovan and Priester (2017) found that motivated reasoning was the proximal influence on forgiveness. This proximal role is reflected in the intercorrelations among the different constructs with forgiveness. In all three studies, motivated reasoning is more closely associated with forgiveness than relationship closeness, desire to maintain the relationship, and empathy^5^.

These correlations provide empirical support for motivated reasoning’s mediational role. They also, however, raise the possibility that motivated reasoning and forgiveness are measures of a single, rather than two different, factors. This is an important point. If these measures are tapping into a single factor, motivated reasoning represents an aspect, rather than an antecedent, of forgiveness (see Fiedler et al., 2011). An inspection of the specific items used to measure motivated reasoning suggests that such an alternative explanation is possible. For example, one of the two motivated reasoning measures used in Study 3 was the extent to which one sees the transgressor in a positive light. It is possible that such perception is an aspect of forgiveness.

To summarize, an alternative explanation to the finding that motivated reasoning, rather than empathy, underlies forgiveness is that the items used to measure motivated reasoning are capturing forgiveness. And as such, proximal mediation is an artifact of the items measuring one construct rather than two distinct constructs.

To best address this alternative explanation, it is ideal to utilize a divergent measure of motivated reasoning that differs sufficiently from the measure of forgiveness so as to provide convergent evidence for the proximal mediational role of motivated reasoning. Recall that motivated reasoning predicts that one’s thoughts, feelings, and reactions are shaped by one’s desire to maintain a relationship; the greater the desire, the more positive and/or less negative the thoughts, feelings, and reactions.

Motivated reasoning, then, is reflected in the valence (i.e., the positivity and/or negativity) of one’s thoughts toward the transgressor and/or the transgression. As such, the valence of thoughts, feelings, and reactions provides a potentially divergent measure of motivated reasoning. That is, instead of (or in addition to) measuring such thoughts, feelings, and reactions through questionnaire items as is typically done, one could have participants provide their own thoughts, feelings, and reactions^6^. Motivated reasoning should be reflected in greater overall positivity and lower overall negativity of such thoughts, feelings, and reactions.

It is worth reflecting upon the use of thoughts as a measure of motivated reasoning. Kunda (1990) clearly conceptualized motivated reasoning as the result of (1) selective retrieval of memories and/or (2) the construction of beliefs. Underlying both of these processes are an individual’s thoughts. As such, a measure of thoughts should reflect the nature of the retrieval and construction processes. And such a measure of thought is provided by the elicitation and measure of cognitive responses (henceforth referred to as thoughts; see Cacioppo et al., 1981). We are not the first to use thoughts as a measure of motivated reasoning (e.g., Harkness et al., 1985; Tetlock and Kim, 1987). Indeed, Murray and Holmes (1997) use such an approach in order to understand the motivated reasoning underlying close relationships.

Based on the conceptualization of and the past use of thoughts to assess motivated reasoning, we adopt such an approach in the present research in order to operationalize motivated reasoning with a measure that differs from the approach used in Donovan and Priester (2017). If such a divergent measure exhibits a similar pattern of proximal mediation, the concern that the results for motivated reasoning are due to it being part of the same construct as forgiveness is mitigated. And as such, support is provided for the influence of motivated reasoning on forgiveness.

In addition to providing a divergent measure, the use of thoughts as a measure of motivated reasoning provides an opportunity for an analysis of the valence of the thoughts. Motivated reasoning might operate by increasing the positive thoughts that one has in reaction to a transgression. Or alternatively, motivated reasoning might operate by decreasing the negative thoughts. Or it may operate by both decreasing negativity and increasing positivity. The use of thoughts to operationalize motivated reasoning allows for an examination of the nature of motivated reasoning in interpersonal forgiveness.

### Analytic Estimation

Although of less concern, a question does exist regarding how to best estimate the two models. The estimation approach used in Donovan and Priester (2017) estimated all possible paths simultaneously. This decision was based in part on the exploratory nature of the research. The research was designed to provide an initial test of the Model of Motivated Interpersonal Forgiveness in addition to comparing its ability to account for interpersonal forgiveness to the empathy model. Since this was the first test between the models, it was possible that other paths might emerge as significant. For example, empathy might have mediated the influence of motivated reasoning on forgiveness, a possibility tested but not supported by the data.

**FIGURE 3:**
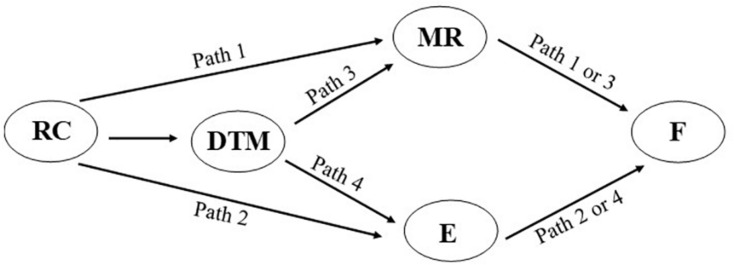
Estimation model specifying the four possible mediational path; Relationship closeness - Motivated Reasoning → Forgiveness (path 1), Relationship closeness → Empathy → Forgiveness (path 2), Relationship closeness → Desire to maintain the Relationship → Motivated Reasoning → Forgiveness (path 3), Relationship closeness → Desire to maintain the Relationship → Empathy → Forgiveness (path 4). RC equals Relationship Closeness, DTM equals Desire to Maintain the Relationship, MR equals Motivated Reasoning, E equals Empathy, and F equals Forgiveness.

One drawback of such an approach in which all possible paths are estimated, however, is that it potentially decreases the ability to detect mediational influences. That is, estimating nonessential paths can decrease the power to detect significance of the essential paths. Such a dilution of power may have contributed to the lack of support for the empathy model of forgiveness. In order to overcome this possibility, a more specific analytic approach was adopted herein, in which only the essential paths associated with each of the two perspectives were tested. This estimation is presented in Figure 3.

Inspection of the figure reveals it tests for the ability of the Model of Motivated Interpersonal Forgiveness and the empathy model without the addition of nonessential paths^7^. Given the importance of desire to maintain the relationship as the process that drives the effect of relationship closeness on forgiveness, it is included as a possible path in the empathy model. As such, the analytic estimation used tests the ability of empathy to play a mediational role for both the influence of relationship closeness on forgiveness (path 2), as well as for the influence of desire to maintain the relationship on forgiveness (path 4). At the same time, the analytic estimation used tests the ability of motivated reasoning to play a mediational role for both the influence of relationship closeness on forgiveness (path 1), as well as for the influence of desire to maintain the relationship on forgiveness (path 3). Again, note that all four of these paths were tested in Donovan and Priester (2017), and only path 3 was found to be significant. However, the current, more focused test allows for greater power to detect the role of empathy in the forgiveness process.

To test these questions, we employed bootstrap OLS regression analyses using a customized mediational model (process v3.4, Hayes, 2018)^8^. The model allows for tests of four possible mediation paths (see Figure 3). The influence of relationship closeness on forgiveness could be mediated by (a) motivated reasoning absent desire to maintain the relationship (path 1), (b) empathy absent desire to maintain the relationship (path 2), (c) desire to maintain the relationship through motivated reasoning (path 3), and/or (d) desire to maintain the relationship through empathy (path 4). In this analysis, it is possible for more than one mediational path to emerge as significant. It is also possible for no mediational paths to emerge as significant.

## Study

The present study was conducted in order to address two concerns. First, and of greatest relevance, are the findings of Donovan and Priester (2017) the result of the motivated reasoning items being conflated with forgiveness? To address this concern, the thoughts, feelings, and reactions during and following the transgression were used to operationalize motivated reasoning in addition to the questionnaire items used in Donovan and Priester (2017). This measure also allows the opportunity to examine whether motivated reasoning operates by reducing the negativity of the thoughts, feelings, and reactions and/or increasing the positivity. Second, and of less relevance, are the results of Donovan and Priester (2017) replicated when a more focused analytic estimation is used to test the relative ability of the two models to explain forgiveness?

### Methods

#### Participants and Procedure

One hundred seven undergraduate students from a West Coast university participated in exchange for partial fulfillment of course credit^9^. Participants were instructed to recall an instance in which a person let them down. Specifically, participants read, “Sometimes people we know let us down. For this study, we would like you to remember a time that a person failed you. Please recall a specific incident when a person hurt and/or disappointed you. This incident can be anything. For example, your friend forgets about an activity you had planned or your significant other cheats on you.” Participants then wrote the name of and relationship with the person. Participants provided a brief description of the incident. Participants then completed two thought-listing tasks and answered a series of questions designed to assess their relationship with the person, desire to maintain the relationship, motivated reasoning, empathy, and forgiveness^10^. This procedure follows that used by Donovan and Priester (2017), the only difference being the inclusion of the thought listing measure.

### Independent and Mediating Variables

#### Relationship Closeness

Relationship closeness has been conceptualized and measured from different perspectives. Perhaps the most commonly used measure for relationship closeness is the Inclusion of the Self in Other Scale (IOS, described below, Aron et al., 1991). We use this measure in the present study. A second approach commonly used is to measure an individual’s feelings of relationship quality and closeness [using measures such as commitment (Rusbult et al., 1991; Arriaga and Agnew, 2001; Tran and Simpson, 2009), loyalty (Fehr, 1988; Fehr and Harasymchuk, 2005), love (Byrne, 1997), and trust (e.g., Luchies et al., 2013)]. We collected and combined these four measures to create a measure of relationship feelings of quality and closeness. The strategy of using two such different measures of relationship closeness was to provide convergent evidence for the influence of relationship closeness. Analyses revealed that the results for the IOS and the second measure were statistically identical. As such, we combined the two measures in order to create an overall relationship closeness measure.

As done in Donovan and Priester (2017), pre-transgression relationship closeness was assessed by these two methods. The first approach utilized the Inclusion of the Other in Self scale (IOS; Aron et al., 1991). The IOS is a scale that comprised seven pairs of circles, which vary in the extent by which they overlap, from only the boundaries touching (equal to one) to complete overlap (equal to seven). Participants were instructed to indicate which pair of circles best represented their relationship. The second approach utilized four items designed to assess relationship feelings of relationship quality and closeness. These items were “I feel that I am committed to this person,” “I consider myself to be highly loyal to this person,” “I love this person,” and “I trust this person.” These four items used 11-point scales anchored with zero equal to “not at all” and 10 equal to “completely.” The four were averaged to create a relationship closeness subscale (α = 0.91). The feelings of relationship quality and closeness measure and the IOS scale were standardized and averaged to create an overall relationship closeness measure (α = 0.83).

#### Desire to Maintain the Relationship

The three items used in Donovan and Priester (2017) were used to measure desire to maintain the relationship. These items were “How motivated were you to restore your relationship with this person?” “I would be really sad if I stopped spending time with this person,” both anchored with zero equal to “not at all” and 10 equal to “completely”; and “I intend to continue interacting with this person,” anchored with zero equal to “disagree” and 10 equal to “agree.” These items were combined in order to create one measure (α = 0.91). Note that the three items reflect (1) motivational, (2) emotional, and (3) intentional components. Results using only the motivational measure provide statistically equivalent results to those obtained using all three.

#### Motivated Reasoning

Motivated reasoning was captured by two methods: thought listings and questionnaire items. For the first, participants listed and coded their own thoughts and feelings related to the transgression. For the second, participants answered motivated reasoning questionnaire items from Donovan and Priester (2017), study 3.

#### Thoughts

In order to elicit a broad profile of thoughts, participants completed two different thought-listing tasks. Each task presented the participants with the instructions at the top of the page, below which were 10 boxes. The first task instructed:

Now, we would like you to take a minute to think about the time the person let you down. We want you to remember how you felt at the time of the incident. What were your thoughts when the person let you down? How did you react? Please answer the following questions:

First, what were your thoughts and feelings when this happened? Please tell us all you can about the incident and how you felt when the incident happened. In each box below, please write one thought or feeling. So, if you have one reaction (thought or feeling), you would use one box. If you have three reactions, you would use three boxes. Use only as many boxes as reactions that you have. You don’t need to use all the boxes. Don’t worry about grammar or complete sentences. Just write enough that it makes sense.

The second task instructed:

In the boxes below, please provide us with your reactions toward this incident. How did you feel about the person following the incident? How did you react? What did you do? Again, use as many boxes as you have reactions.

After writing their thoughts, participants coded each thought as to whether it was positive, negative, or neutral. To assess the extent to which motivated reasoning influenced forgiveness, two measures were constructed. The first examined the degree to which motivated reasoning buffered against negative interpretation of the incident. To do so, a measure was created by summing the negative thoughts from each thought-listing task. A second measure examined the degree to which motivated reasoning created a positive interpretation of the incident. To do so, a measure was created by summing the positive thoughts from each thought-listing task.

#### Motivated Reasoning Questionnaire Items

The two items used in Donovan and Priester (2017) were used to operationalize motivated reasoning^11^. These items were “I believe that the next time I interact with this person, they will live up to my expectations” and “I view this person in a positive light.” Both items were measured on 11-point scales anchored with zero equal to “not at all” and 10 equal to “completely.” These two items were averaged to create the motivated reasoning measure (α = 0.78).

#### Empathy

Empathy was measured using two items from the index of empathetic concern (Coke et al., 1978; Exline and Zell, 2009; Fehr et al., 2010): “I felt empathetic toward the person following the incident” and “I felt compassionate toward the person following the incident.” Both items were assessed by 11-point scales, anchored with zero equal to “not at all” and 10 equal to “completely.” These two items were averaged in order to create a measure of empathy (α = 0.80).

### Dependent Variable

#### Forgiveness

Forgiveness was assessed by three items: “I have forgiven the person following the incident,” “I want to avoid the person” (reverse coded), and “I want to take revenge on the person” (reverse coded). The three items were assessed by 11-point scales anchored with zero equal to “not at all” and 10 equal to “completely.” The items were averaged to create a measure of forgiveness (α = 0.70)^12^.

## Results

### Univariate Statistics and Relationships Among Variables

The univariate statistics for each variable and the correlations among the variables are presented in Table 1. Of note is that the measures of skewness and kurtosis skewness reflect that the variables are normally distributed as they fall within the range of −1 and 1. This was not the case, however, for positive thoughts^13^. Upon the recommendation of a reviewer, the data for positive thoughts were corrected by removing three responses that were beyond two standard deviations (SDs) of the mean^14^. The results using the corrected positive measure are reported. Use of the uncorrected positive measure reveals a non-significant influence of positive thoughts on forgiveness.

**TABLE 1:** Univariate statistics and correlations.

Item	Measures	Mean	Skewness	Kurtosis	1	2	3	4	5	6	7
1	Forgiveness	6.59	−0.63	−0.26	–						
2	Closeness	0.00	0.02	−0.95	0.48	–					
					[<0.0001]						
3	DTM	5.33	−0.25	−1.21	0.64	0.83	–				
					[<0.0001]	[<0.0001]					
4	MR Negative Thoughts	4.87	−0.03	0.12	−0.33	−0.08	−0.21	–			
					[0.0005]	[0.43]	[0.03]				
5	MR Positive Thoughts	0.67	3.09	12.69	0.1	−0.07	0.01	−0.3	–		
					[0.31]	[0.5]	[0.88]	[0.002]			
6	MR Questionnaire Items	5.30	−0.27	−0.91	0.72	0.69	0.79	−0.31	0.1	–	
					[<0.0001]	[<0.0001]	[<0.0001]	[0.0013]	[0.3262]		
7	Empathy	2.89	0.57	−0.14	0.22	0.31	0.28	−0.1	0.19	0.29	–
					[0.02]	[0.001]	0[.004]	[0.29]	[0.05]	[0.003]	

### Independent Predictors of Forgiveness

Forgiveness (F) was regressed on relationship closeness (RC), desire to maintain the relationship (DTM), thought negativity, thought positivity, motivated reasoning questionnaire items (MRQI), and empathy (E). Replicating prior empirical results, relationship closeness, desire to maintain the relationship, and empathy all significantly predicted forgiveness: *b* = 1.48, *F*(106) = 30.60, *p* < 0.0001 (RC); *b* = 0.53, *F*(106) = 71.37, *p* < 0.0001 (DTM); *b* = 0.66, *F*(106) = 115.00, *p* < 0.0001 (MRQI); *b* = 0.25, *F*(106) = 5.66, *p* = 0.02 (E). Analyses of the thought listing data revealed that both thought negativity (*b* = −0.49, *F*(106) = 13.11, *p* = 0.0005) and positivity (*b* = 0.84, *F*(106) = 6.03, *p* = 0.0158) significantly predicted forgiveness.

### Model Analysis Strategy

Recall that the present study was conducted to replicate and extend the results of Donovan and Priester (2017). The first extension concerns the nature of motivated reasoning: Do the results extend to a divergent measure of motivated reasoning? The second extension concerns the nature of the estimation used to test the two models: Do the results extend to an estimation in which just the focal paths are estimated, or instead does empathy emerge as a significant mediator of forgiveness?

**FIGURE 4:**
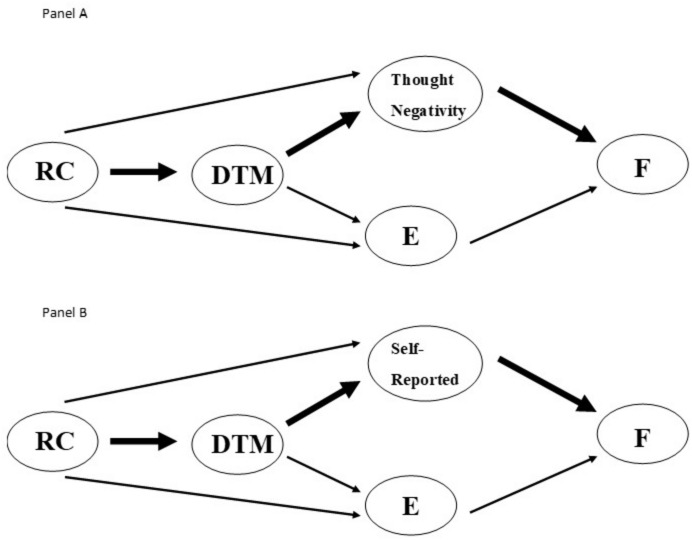
Estimation results. **Panel A** Depicts the model using thought negativity for Motivated Reasoning. **Panel B** Depicts the models using self-reported Motivated Reasoning. RC equals Relationship Closeness, DTM equals Desire to Maintain the Relationship, MR equals Motivated Reasoning, E equals Empathy, and F equals Forgiveness.

As explicated above, we employed bootstrap OLS regression analyses using a customized mediational model (process v3.4, Hayes, 2018)^15^. Such a model allows for the test of four possible mediation paths (Figure 3). The results of such an analysis produce an upper and lower confidence interval for each of the four possible mediational paths. Paths in which the confidence intervals do not include zero indicate that the path is significant. The confidence intervals for all possible paths are included in Table 2, and the results are depicted in Figure 4. For ease of representation, the significant paths are designated in bold in both the table and figure.

**TABLE 2:** Model estimation and comparison results.

			Bootstrap 95% CI	
Mediation models	Estimate	SE	Lower CI	Upper CI
Paths Panel a, MR = Thought Negativity				
RC → Thought Negativity → F	−0.29	−0.22	−0.83	0.03
RC → CE → F	0.05	0.09	−0.13	0.23
**RC → DTM → Thought Negativity → F**	**0.36**	**0.21**	**0.06**	**0.88**
RC → DTM → CE → F	0.01	0.05	−0.07	0.15
**Paths Panel b, MR = Questionnaire Items**				
RC → Questionnaire Items → F	0.27	0.29	−0.27	0.89
RC → CE → F	0.02	0.08	−0.14	0.18
**RC → DTM → Questionnaire Items → F**	**1.35**	**0.32**	**0.68**	**1.93**
RC → DTM → CE → F	0.01	0.04	−0.08	0.09

*Bold is significant.*

### Thoughts

We conducted two analyses for thoughts as a potential mediator: one using thought positivity and one using thought negativity.

### Motivated Reasoning Thought Negativity

The use of thought negativity as an operationalization of motivated reasoning replicated and extended past results. Specifically, the mediation path in which relationship closeness → desire to maintain the relationship → thought negativity → forgiveness (path 3) did not include zero (lower confidence interval = 0.06, upper confidence interval = 0.88), and as such, is significant. In contrast, none of the other three mediational paths is significant, in that their confidence intervals all include zero: path 1 (lower confidence interval = −0.83, upper confidence interval = 0.03), path 2 (lower confidence interval = −0.13, upper confidence interval = 0.23), and path 4 (lower confidence interval = −0.07, upper confidence interval = 0.15). These results are presented in Figure 4A and Table 2A.

### Motivated Reasoning Thought Positivity

The use of thought positivity as an operationalization of motivated reasoning yielded no significant mediation paths.

### Motivated Reasoning Questionnaire Item

The use of questionnaire items to operationalize motivated reasoning replicated the results of Donovan and Priester (2017). Specifically, the mediation path in which relationship closeness → desire to maintain the relationship → questionnaire items → forgiveness (path 3) did not include zero (lower confidence interval = 0.68, upper confidence interval = 1.93), and as such, is significant. In contrast, none of the other three mediational paths is significant, in that their confidence intervals all include zero: path 1 (lower confidence interval = −0.27, upper confidence interval = 0.89), path 2 (lower confidence interval = −0.14, upper confidence interval = 0.18), and path 4 (lower confidence interval = −0.08, upper confidence interval = 0.09). These results are presented in Figure 4B and Table 2B.

## General Discussion

The present study was conducted in order to explore two questions that emerged from the empirical support for the Model of Motivated Interpersonal Forgiveness. Both concerns, at the most basic, were to what extent the measures and analyses used by Donovan and Priester (2017) reduced the ability to detect the mediational influence of empathy on interpersonal forgiveness. The present study was conducted in order to address these questions in order to better be able to find a possible mediational role of empathy on interpersonal forgiveness.

### Addressing the Two Questions

#### Analytic Estimation

One question emerged from consideration of the analytic estimation used to test between the two models. In short, did the inclusion of nonfocal paths reduce the power to observe the mediational influence of empathy on forgiveness? To address this concern, a more focused estimation was used, in which only the focal paths were estimated. The Model of Motivated Interpersonal Forgiveness was replicated using this modified estimation approach. No paths that included empathy emerged as significant, suggesting that the analytic estimation used in Donovan and Priester (2017) did not account for the lack of support for the empathy model of forgiveness. While empathy was a significant independent predictor of forgiveness, even with the estimation of only essential paths, it did not emerge as a significant mediator of forgiveness. The analytic estimation used tests the ability of empathy to play a mediational role for both the influence of relationship closeness on forgiveness (path 2), as well as for the influence of desire to maintain the relationship on forgiveness (path 4), and neither emerged as significant. Thus, empathy is related to forgiveness, but it is does not mediate between relationship closeness, or relationship closeness and desire to maintain the relationship, and forgiveness.

#### Measure of Motivated Reasoning

A second question emerged from consideration of the measure used to capture motivated reasoning. Specifically, did the measure tap into forgiveness as well as motivated reasoning? The current research operationalized motivated reasoning by measuring the thoughts, feelings, and reactions that individuals had in relation to a relationship transgression through a thought-listing procedure, in addition to using more standard questionnaire items. The Model of Motivated Interpersonal Forgiveness was replicated using negative thoughts as a measure of motivated reasoning. No paths that included empathy emerged as significant, suggesting that the specific measure of motivated reasoning used in Donovan and Priester (2017) did not account of the lack of support for the empathy model of forgiveness.

### The Nature of Motivated Reasoning

The use of thoughts as an operationalization of motivated reasoning also allowed for insight into the nature of motivated reasoning in interpersonal forgiveness. *A priori*, it was unknown as to whether motivated reasoning would consist of fewer negative thoughts and/or more positive thoughts. Although both thought positivity and negativity significantly predicted forgiveness, only thought negativity served as the most proximal mediator to forgiveness.

These results suggest that the power of motivated reasoning, at least within the context of interpersonal forgiveness, comes from a less negative, rather than a more positive, interpretation of the transgression.

Such a finding may help to integrate interpersonal motivated reasoning within a broader theoretical framework. In general, it has been found that negative information and events have a more powerful influence on physiological, cognitive, emotional, and social responses than positive events (see, for example, Taylor, 1991; Ito et al., 1998). Interestingly, the current findings suggest that motivated reasoning shapes the perception of transgressions to be less negative by buffering the negative resulting thoughts. And as such, understanding a transgression to be less negative may be an especially powerful process by which to foster forgiveness.

This finding raises intriguing questions regarding motivated reasoning processes. One conceptualization of motivated reasoning used in the present research (as well as Donovan and Priester, 2017) is positive illusions (e.g., Murray and Holmes, 1997, 1999; Carswell et al., 2019). The conceptualization of motivated reasoning as positive illusions leads to an intuition that such illusion emerges through increases in positivity by means of increased positive thoughts. However, the current finding suggests that positive illusions may well emerge through decreases in negativity by means of fewer negative thoughts. The partners are still perceived to be relatively more positive. It is just that this occurs because they are perceived less negatively, rather than more positively. Of course, we find this reduction of negative thoughts in the domain of interpersonal forgiveness. An interesting question arises as to whether this buffering effect is restricted to instances of transgressions or instead extends to other interpersonal interactions and outcomes.

### The Importance of Desire to Maintain a Relationship

The present research reaffirms the importance of desire to maintain the relationship. Desire to maintain the relationship consistently mediates the influence of relationship closeness on the downstream variables of motivated reasoning and forgiveness. Two theoretical questions emerge. First, to what extent does desire to maintain the relationship mediate the effects of relationship closeness beyond interpersonal forgiveness? For example, is it desire to maintain a relationship that mediates the influence of relationship closeness on other relationship processes and outcomes? Second, to what extent might desire to maintain the relationship provide a common causal mechanism (i.e., act as a mediator) for relationship constructs beyond relationship closeness, such as commitment, satisfaction, trust, and love. The present research raises the question of whether these disparate constructs may all share the property of operating through desire to maintain the relationship. If so, such desire may provide a unifying lens through which to conceptualize relations in general.

## Future Research and Limitations

However, the current results should be generalized with caution. This is the first finding to suggest that thought negativity is a more powerful aspect of motivated reasoning than positivity in influencing forgiveness. And given that both thought negativity and positivity significantly influenced forgiveness, it is possible that, with a more powerful study, thought positivity might begin to exhibit a mediational influence similar to negativity. As such, further investigation is warranted before making definitive inferences as to the relative role of negative versus positivity thoughts.

Consider that forgiveness has been shown to be positively associated with many beneficial constructs: psychological well-being (Karremans et al., 2003; Pareek et al., 2016; Akhtar and Barlow, 2018; Barcaccia et al., 2019), physical health (Lee and Enright, 2019), decreased blood pressure for both victim and perpetrator (Hannon et al., 2010), greater health resilience (Worthington and Scherer, 2004), increased longevity [e.g., Barcaccia et al. (2020); see Witvliet et al. (2001)], and reduced depression (Toussaint et al., 2012). It is also important to consider, however, that forgiveness may not always be the ideal outcome following a transgression. Consider spousal abuse. Victims of such abuse could forgive, only to re-experience similar, or worse, abuse in the future (Miller and Porter, 1983; Shaver and Drown, 1986; Kearns and Fincham, 2004). In the future, researchers could explore the implications of a reduction in negative thoughts across different relationship dynamics to better understand when and why partners remain in abusive relationships.

A limitation and concern that could be explored in future research is that these models have been tested on both recall and hypothetical scenarios (Donovan and Priester, 2017), which ameliorate the concerns of either method on its own. However, a longitudinal study would allow measurement of the constructs across time, which would afford the opportunity to better test the sequential order hypothesized by the model (e.g., Murray and Holmes, 1997; Finkel et al., 2002; Orth et al., 2008).

## Summary

The current research provides additional support for the Model of Motivated Interpersonal Forgiveness. The present research suggests that the findings of Donovan and Priester (2017) do not appear to be the result of analytic estimation or measurement issues. Rather, the Model of Motivated Interpersonal Forgiveness appears to provide a compelling framework by which to understand the psychological process through which interpersonal forgiveness emerges. Specifically, the model provides answers to when, why, and how interpersonal forgiveness emerges.

## Data Availability Statement

The datasets generated for this study are available on request to the corresponding author.

## Ethics Statement

All data collection involving human participants were reviewed and approved by Institutional Review Board of University of Southern California. The participants provided their written informed consent to participate in this study.

## Author Contributions

Both authors listed have made a substantial, direct and intellectual contribution to the work, and approved it for publication.

## Conflict of Interest

The authors declare that the research was conducted in the absence of any commercial or financial relationships that could be construed as a potential conflict of interest.

## Footnotes

1 Web of science search on December 30, 2019 with topic equal to forgiveness.

2 Other research has found evidence consistent with the notion that motivated reasoning as instantiated by perception of a transgressor mediates forgiveness (e.g., Hook et al., 2015).

3 Specifically, McCullough et al. (1997) define forgiveness: We define *interpersonal forgiving* as the set of motivational changes whereby one becomes (a) decreasingly motivated to retaliate against an offending relationship partner, (b) decreasingly motivated to maintain estrangement from the offender, and (c) increasingly motivated by conciliation and goodwill for the offender, despite the offender’s hurtful actions.

4 These paths included (a) relationship → empathy → forgiveness, (b) relationship → desire to maintain the relationship → empathy → forgiveness, (c) relationship → motivated reasoning → empathy → forgiveness, and (d) relationship → desire to maintain the relationship → motivated reasoning → empathy → forgiveness.

5 The correlations between motivated reasoning and forgiveness range from *r* = 0.67 (study 1), *r* = 0.89 (study 2), to *r* = 0.45 (study 3).

6 We base our approach upon that used within the field of attitudes and persuasion. In these studies, the idiosyncratic cognitive responses of individuals are often assessed. Individuals are asked to write their thoughts and feelings toward an attitude object, after which participants code their own thoughts and feelings, typically as to whether the thoughts are positive, negative, or neutral (see Cacioppo and Petty, 1982).

7 The specific paths not tested are as follows: relationship closeness → desire to maintain the relationship → forgiveness, relationship closeness → motivated reasoning → empathy → forgiveness, and relationship closeness → desire to maintain the relationship → motivated reasoning → empathy → forgiveness.

8 To construct this estimation, the bmatrix was set to 1 1 1 1 1 0 1 0 1 1.

9 Sample size was determined prior to data collection, and no additional data were collected following analyses. Sample size was based on the sample sizes used in Donovan and Priester (2017). Specifically, study 2 of Donovan and Priester collected data from 120 participants. We attempted to collect data from the same number.

10 Other, nonfocal questions were also assessed. Of particular interest, we collected the tendency to forgive scale (Brown, 2003). Tendency to forgive was associated with greater forgiveness (*b* = 0.73, *F*(1,105) = 7.9, *p* = 0.0058). However, it did not interact with any other variables and is thus not considered further.

11 As was done in Study 3 of Donovan and Priester, an array of additional motivated reasoning items was collected. The results using a measure of motivated reasoning created by combining these items replicated the results of Donovan and Priester (2017) and were statistically equivalent to the results reported herein. As such, they are not discussed further.

12 The results of using just the single-item measure and just the revenge/avoidance scale were statistically identical. That is, the results using either approach by itself produced results similar to each other, as well as to the results using the combined measure.

13 For positive thoughts, the uncorrected univariate measures are mean = 0.67, SD = 1.27, skewness = 3.0943, and kurtosis = 12.6885.

14 These responses were 4, 6, and 8.

15 To construct this estimation, the bmatrix was set to 1 1 1 1 1 0 1 0 1 1.
